# Factors Associated with the Odds, Duration, and Costs of Health-Related Absenteeism: A Population-Based Study in São Paulo City, Brazil

**DOI:** 10.3390/healthcare14101260

**Published:** 2026-05-07

**Authors:** Lucas Akio Iza Trindade, Jaqueline Lopes Pereira, Marcelo Macedo Rogero, Regina Mara Fisberg, Flavia Mori Sarti

**Affiliations:** 1 School of Public Health, University of São Paulo, São Paulo 01246-904, Brazil; jaque.lps@gmail.com (J.L.P.); mmrogero@usp.br (M.M.R.); rfisberg@usp.br (R.M.F.); 2 School of Arts, Sciences and Humanities, University of São Paulo, São Paulo 03828-000, Brazil; flamori@usp.br

**Keywords:** sick leave, indirect costs, non-communicable diseases, lifestyle factors, occupational health, Brazil

## Abstract

**Highlights:**

**What are the main findings?**

- Cardiovascular diseases, type 2 diabetes, and tobacco use significantly increase both the odds and duration of sick leave.
- Meeting recommended levels of leisure-time physical activity is associated with lower indirect costs from health-related absenteeism.

**What are the implications of the main findings?**

- Public health strategies promoting physical activity and smoking cessation may reduce economic losses in productivity.
- Estimating indirect costs provides evidence to prioritize preventive interventions in middle-income countries, such as Brazil.

**Abstract:**

**Background/Objectives:** Non-Communicable Diseases (NCD) impose a substantial socioeconomic burden on health systems through direct costs and indirect costs from productivity loss due to health-related absenteeism. While lifestyle factors are crucial for NCD prevention, evidence regarding their association with absenteeism in middle-income countries, such as Brazil, remains limited. In this context, the present study aims to analyze factors associated with the odds, duration, and costs of health-related absenteeism in São Paulo City, Brazil. **Methods:** Quantitative analysis was performed using microdata from the São Paulo Health Survey 2003, 2008, and 2015 (ISA-Capital). Logistic and negative binomial regression models identified factors associated with the odds and duration of health-related absenteeism. The human capital approach was used to estimate indirect costs (Int$ PPP), while two-part regression models (logit and generalized linear model) and average marginal effects (ME) identified cost-associated factors. **Results:** Tobacco use and NCD diagnoses (hypertension, type 2 diabetes, and cardiovascular diseases) significantly increased the odds and duration of absenteeism. Conversely, meeting recommended leisure-time physical activity levels was associated with lower indirect costs (ME = −33.94, *p* < 0.05). Higher costs were significantly driven by tobacco use (ME = 48.68, *p* < 0.01) and NCD, namely cardiovascular diseases (ME = 62.73), diabetes (ME = 55.18), hypertension (ME = 52.13), and obesity (ME = 36.45), all with *p* < 0.05. **Conclusions:** Promoting leisure-time physical activity and tobacco cessation may be important strategies for public health policies aiming to enhance productivity by reducing the frequency, duration, and economic burden of health-related absenteeism, complementing the necessary diagnosis and monitoring of NCD.

## 1. Introduction

The current epidemiological scenario in Brazil and other middle-income countries is marked by an increase in the incidence and prevalence of cardiometabolic risk factors and associated non-communicable diseases (NCDs) [1,2]. High blood pressure (HBP), obesity, type 2 diabetes (T2D), and cardiovascular diseases (CVD) may cause multimorbidity in individuals and are based on the interconnection of modifiable and non-modifiable biological, environmental, and behavioral factors [3].

Unhealthy lifestyle patterns, like physical inactivity or insufficient physical activity, sedentary behavior, and smoking, are associated with increased risk for the occurrence of cardiometabolic risk factors and related NCDs [4,5,6]. Highlighting the complex relationships between health behaviors and cardiometabolic health may be an important tool for health promotion and disease prevention. Individuals usually present co-occurrence of several risky or healthy lifestyle factors, resulting in interdependent and synergistic effects on the cardiometabolic profile [7].

Therefore, modifiable factors, such as health behaviors and some cardiometabolic risk factors, may be influenced by health policies aimed at preventing and controlling the onset and progression of NCDs [7]. Additionally, NCDs impose a significant socioeconomic burden on national health systems in low- and middle-income countries [8]. In the Brazilian context, cardiometabolic risk factors and related NCDs are associated with the risk of impoverishment due to catastrophic health expenditures derived from the need to pay for health services and products [9].

Beyond direct costs, indirect costs arising from health-related absenteeism and other productivity losses should be acknowledged for their substantial impact on individuals’ livelihoods and well-being [10]. In Brazil, NCDs have a major influence on health-related absenteeism among the economically active population. Cardiometabolic risk factors, such as high blood pressure and dyslipidemia, are among the main causes of absenteeism related to illness [11]. Thus, lifestyle factors may also play a significant role in preventing health-related absenteeism and its associated costs in Brazil, as observed in high-income countries [12].

However, evidence on the associations among lifestyle factors, health-related absenteeism, and its related costs remains underexplored at the population level in middle-income countries. In Brazil, the research gap may stem from limited population surveys and administrative databases that support economic valuation, as well as from a lack of comprehensive data on absenteeism and biological and lifestyle factors. Previous studies in Brazil have analyzed the association between health-related absenteeism and lifestyle factors using convenience samples, which are not representative of the population [13,14]. Thus, there is insufficient evidence to support evidence-based decision-making in public policies aimed at minimizing the socioeconomic burden of productivity losses from the perspectives of individuals, households, and companies. Furthermore, effective data-driven strategies may increase productivity in middle-income countries by improving workers’ health.

Health-related absenteeism comprises a complex phenomenon involving multiple intertwining determinants [15]. It is therefore critical to identify modifiable determinants associated with the odds of health-related absenteeism, its duration, and its costs. These dimensions of absenteeism, though interrelated, may respond to distinct factors that public policy strategies for health promotion could address: while clinical conditions and lifestyle may dominate in driving absenteeism odds and duration, the analysis of costs incorporates socioeconomic heterogeneities, providing a broader quantification of the economic burden and productivity loss at the population level. Thus, examining these interrelated yet distinct dimensions may provide evidence to help policymakers tailor interventions to address absenteeism holistically [15].

Therefore, the present study aims to analyze factors associated with the odds and duration of health-related absenteeism, and to investigate determinants of health-related absenteeism costs. The study particularly focuses on the associations of lifestyle factors (leisure-time physical activity, sedentary behavior, and tobacco use), cardiometabolic risk factors, and associated NCDs (high blood pressure, obesity, type 2 diabetes, and cardiovascular diseases) in a representative sample of the population of São Paulo City, Brazil.

## 2. Materials and Methods

### 2.1. Study Design

This is a cross-sectional observational study with quantitative analysis of microdata from the São Paulo Health Survey (ISA-Capital), a population-representative survey for the city of São Paulo, Brazil. The target population for the present study is the economically active population who reported formal employment or paid work; therefore, the population is susceptible to health-related absenteeism.

The city of São Paulo has been Brazil’s largest city and urban center since the mid-1960s, presenting a population of approximately 11.5 million individuals in 2022 [16]. The ISA-Capital is a population-based health survey that collects data on health conditions, lifestyle, and use of health services, as well as household, demographic, and socioeconomic information from individuals living in private and permanent households in the urban area of São Paulo.

There were three editions of the ISA-Capital, held in 2003, 2008, and 2015, in collaboration with researchers from the University of São Paulo and the São Paulo City Health Department. In all three editions, the population sampling process was probabilistic, in two stages, with a random selection of census tracts and households. Individuals living in the randomly selected households were invited to take part in the survey. Subsequently, sampling weights, regarding the inverse probability of inclusion in the sample, and further adjusted for non-response, were applied to the interviewed individuals to ensure population-level representativeness; details regarding the calculation of the sampling weights used in the ISA-Capital have been previously published [17].

Data and information were collected through interviews with participating individuals, using a semi-structured questionnaire administered by previously trained research agents. Most of the questions in the questionnaires were closed-ended and based on previously tested and validated questionnaires for the Brazilian population. Further details on the sampling process and survey instruments have been previously published [17,18].

### 2.2. Dataset

The dataset for this study includes microdata from the three editions of the ISA-Capital. At first, the databases were analyzed individually to select variables that were directly comparable between all the editions. Secondly, the three databases, already individually treated, were combined into a single dataset, enabling statistical analysis across the entire period of 2003–2015, comprising a sample of 6595 individuals.

Variables that were not directly comparable across the three survey editions were excluded from the analyses to avoid bias in statistical estimates, either because they were not collected in one of the survey editions or because of significant changes in the questionnaire wording. Observations missed due to individuals refusing to answer (n = 91, approximately 1.4% of the present study sample) were also excluded from the database. Therefore, only individuals with complete information were included in the present study (n = 6504).

Given the target population of the present study, data from individuals < 16 years old were excluded from the dataset, as the minimum age for formal employment is 16 years under the 1988 Brazilian Federal Constitution [19]. Data from individuals ≥ 16 years old or older included in this study correspond to individuals who report formal employment or paid work.

Thus, the sample for this study includes data on 1654 individuals from the 2003 ISA-Capital, 2081 individuals from the 2008 ISA-Capital, and 2769 individuals from the 2015 ISA-Capital, totaling 6504 economically active individuals for the period 2003–2015. The three waves of the ISA-Capital survey were pooled while preserving the complexity of the sampling design. Indicators for primary sampling units and sampling strata were individualized for each survey edition, and sampling weights were rescaled to ensure population-level representativeness for the city of São Paulo [20]. This enabled the conduction of statistical analyses on the study’s analytical database, covering the 2003–2015 period.

### 2.3. Variables

#### 2.3.1. Outcome Variables

The study’s outcome variables are health-related absenteeism, the duration of health-related absenteeism (work days missed), and the costs of health-related absenteeism (monetary value of productivity losses). The absenteeism variable refers to self-reported absences from work due to health-related reasons, including external causes of morbidity (e.g., accidents), in the 12 months preceding the survey date. The variable referring to absenteeism was collected in response to the following question: “How many days did you have to be absent from work, school, or routine activities?” In addition, the questionnaire considers episodes of disabling health problems in the month before the survey date and hospital admissions in the 12 months before the survey date.

Although the wording of this question encompasses school and routine activities, restricting our analytical sample exclusively to economically active adults (i.e., those engaged in formal employment or paid work) allowed us to use this variable as a proxy for work-related absenteeism.

Individuals who reported at least one day of absence were considered exposed to health-related absenteeism, according to a categorical variable (1 = yes/0 = no). The number of work days missed reported by individuals was then treated as a discrete variable (the duration of health-related absenteeism). The estimation of health-related absenteeism costs was based on productivity losses. The costs were estimated using the human capital approach [10], following the guidelines of the Brazilian Ministry of Health [21], based on the opportunity cost of labor days lost due to illness, using the individual’s annual wage per working day as a proxy for productivity.

To calculate the annual wage, we used a variable referring to the monthly wage declared by the individual in response to the following question in the survey: “What was your gross income last month from your main job?” The annual salary is the monthly salary multiplied by 12. Subsequently, the wage rate per working day was calculated as the annual salary divided by the number of working days in the year before the interview, excluding weekends and holidays.

Thus, the cost of absenteeism refers to the product of the wage rate per working day by the number of work days missed due to health-related absenteeism, as summarized in Equation (1):

(1) $$C_{it}=d_{it}\times w_{it}$$

where $C_{it}$ = costs of health-related absenteeism for individual *i* in period *t* (survey years); $d_{it}$ = days absent from work due to illness for individual *i* in period *t*; and $w_{it}$ = wage rate per working day for individual *i* in period *t*.

Costs of health-related absenteeism were updated to December 2015 (the year of the last edition of the ISA-Capital survey) using the Broad Consumer Price Index (IPCA), defined by the Brazilian Institute of Geography and Statistics (IBGE) [22]. Subsequently, the costs were converted to international purchasing power parity (PPP) units using the World Bank’s 2015 conversion factor [23].

It is important to emphasize that, beyond analyzing the odds and duration of health-related absenteeism, estimating its costs through the human-capital approach integrates the socioeconomic and health aspects of the individuals; therefore, the same duration of sick leave may result in a different economic burden [10]. The cost analysis is essential to quantify productivity losses and to understand factors associated with the economic burden of health-related absenteeism in a population context, providing evidence for decision-making in public health policies.

#### 2.3.2. Variables of Interest

The variables of interest refer to the health and lifestyle characteristics of the individuals. Health characteristics include cardiometabolic risk factors and medical diagnosis of NCDs: cardiovascular diseases, high blood pressure, and type 2 diabetes. Lifestyle characteristics include tobacco use, leisure-time physical activity, and sedentary behavior.

The NCDs medical diagnosis variables were self-reported by individuals, based on physicians’ formal diagnoses. All variables are binary, with 1 indicating the presence of the diagnosis and 0 indicating its absence (1 = yes/0 = no). The diagnosis of cardiovascular diseases includes the following conditions: angina, coronary heart disease, arrhythmia, myocardial infarction, and other heart diseases.

Obesity was determined from the weight and height reported by individuals during personal interviews. Subsequently, the Body Mass Index (BMI) was calculated as body weight (in kilograms) divided by height squared (in meters) and categorized according to WHO recommendations [24]. Individuals with obesity were adults with a BMI ≥ 30.0 kg/m^2^ and adolescents with a z-score > 2 units above the WHO child growth reference curves [25]. Further studies analyzed and evaluated the information reported by individuals participating in the ISA-Capital surveys on anthropometric characteristics, including biological sex, age, physical activity level, and cardiometabolic risk factors [26].

Regarding lifestyle variables, smoking was considered based on the daily habit of smoking tobacco products, regardless of the number of cigarettes consumed. Sedentary behavior refers to the time spent sitting during the week, especially during leisure time (watching television and using computers/tablets) and during work or study. Time spent in a sitting position during transportation was not considered in the calculation. Given the lack of international consensus on cut-off points for sedentarism and the risk of developing NCDs, individuals in the upper tertile of time spent sitting who did not meet the recommendations for leisure-time physical activity were considered sedentary [27].

Leisure-time physical activity was estimated using the long version of the International Physical Activity Questionnaire (IPAQ) [28], translated and validated for the Brazilian population [29]. Physical activity was measured as the time spent in moderate-to-vigorous physical activity during leisure time each week. Individuals under 18 who reported 300 min or more, and adults and older people who reported 150 min or more, were considered compliant with WHO recommendations [4,27].

All lifestyle variables are binary, with values of 1 indicating the occurrence of the health behavior and 0 indicating its absence (1 = yes/0 = no).

#### 2.3.3. Variables of Control

The control variables correspond to the demographic and socioeconomic characteristics of the individuals, and the characteristics of the survey:

- Demographic characteristics: age (discrete variable); skin color/ethnicity (1 = white/0 = black, brown, indigenous, yellow); marital status (1 = married or civil union/0 = single, divorced or widowed).
- Socioeconomic characteristics: per capita household income (used as a continuous variable solely for descriptive sample characterization, and categorized into low-, middle-, and high-income tertiles for the regression models); private health insurance (1 = yes/0 = no); university degree (1 = equal or higher than university/0 = lower than university).
- Survey characteristics: years of the ISA-Capital surveys (2003, 2008, or 2015).

The age bracket of individuals included in the study corresponds to the economically active population, i.e., individuals aged ≥16 years who reported formal employment or paid work in the survey reference period. Individuals < 16 years were excluded from the study due to the minimum age for formal employment established by the 1988 Federal Constitution of Brazil [19].

The household income per capita corresponds to the ratio of the sum of all the incomes of the individuals living in the household divided by the total number of residents. The values were updated using the IPCA for December 2015 and then converted into international PPP units. To preserve non-linear socioeconomic effects in the multiple regression models, per capita household income was categorized into low, middle, and high-income levels based on sample tertiles. Table 1 describes the variables in the study.

### 2.4. Statistical Analysis

The study used two empirical strategies. The first strategy examined factors related to the odds and duration of health-related absenteeism. The second strategy focused on analyzing factors associated with health-related absenteeism costs.

Logistic regression and negative binomial regression models were estimated for the first strategy, allowing the calculation of odds ratios (OR) for the odds of health-related absenteeism and incidence rate ratios (IRR) for the number of work days missed due to health-related absenteeism. Model estimation allows observation of risk and protective factors associated with the occurrence and duration of health-related absenteeism.

The second strategy was to estimate a two-part model to analyze factors associated with the costs of health-related absenteeism, using a logit regression (first part) and a generalized linear model (second part, gamma distribution with a log link function). This strategy enabled us to estimate coefficients for health-related absenteeism costs. Subsequently, average marginal effects were estimated from both models, indicating the coefficients’ contributions to the costs.

The empirical strategies adopted consider the relatively low frequency of health-related absenteeism at the population level, allowing for the modeling of data with distributions that contain an excess of zeros, meaning absence of health-related absenteeism, and improving the fit to the data by avoiding the interference of excess zeros in the estimators [30,31,32].

In addition, it is important to emphasize that adopting multivariate analysis accounts for the complex relationships between lifestyle and cardiometabolic risk factors. These have interdependent characteristics and synergistic effects on overall health status [3,7]; hence, the importance of considering analytical strategies that can control each factor’s effects on the odds, duration, and costs of health-related absenteeism. To address this complex interplay, our primary analytical strategy aimed to estimate mutually adjusted associations. Although these variables may occupy different positions on the same inferential pathway (e.g., lifestyle influencing cardiometabolic risks, which in turn lead to clinical diagnoses), including them simultaneously allows us to assess the independent, direct contribution of each factor to health-related absenteeism outcomes. This approach models the broader risk profile commonly seen in clinical and occupational practice, where individuals present with a co-occurrence of these conditions [3,7].

Regarding potential mediation effects, we conducted a sensitivity analysis using a block-wise modeling approach [33], sequentially introducing lifestyle factors and health characteristics. The results are presented in the Supplementary Materials (Tables S1–S3c). Furthermore, considering potential multicollinearity among the variables of interest and control in the multiple models [34]. variance inflation factors (VIF) and a Spearman rank correlation matrix were estimated. The results for variables included in the models are presented in the Supplementary Materials (Tables S3 and S4).

All statistical analyses, including descriptive statistics, logistic and negative binomial regression, and the two-part model, were conducted using Stata^®^ software (StataCorp., College Station, TX, USA), version 18.0. The analysis incorporated the ISA-Capital’s complex survey design features (weights, strata and primary sample units), ensuring representativeness at the population level, and including adjustments for potential correlations between subgroups of the sample, with a statistical significance level of 5% (*p*-value < 0.05).

### 2.5. Ethical Aspects

The three editions of the ISA-Capital 2003, 2008, and 2015 surveys were evaluated and approved by the Research Ethics Committee of the School of Public Health of the University of São Paulo (respectively: CAAE 32344014.3.0000.5421; 36607614.5.0000.5421) and by the Municipal Health Department of the City of São Paulo (CAAE 32344014.3.3001.0086). Informed consent was obtained from the survey participants in accordance with the ethical principles of the Declaration of Helsinki. The present study was also evaluated and approved by the Research Ethics Committee of the School of Public Health of the University of São Paulo (CAAE 48271721.4.0000.5421).

## 3. Results

### 3.1. Descriptive Analysis

Most participants in the study sample were female, self-identified as white, and married. Few participants reported private health insurance or a university degree or higher. The average age was approximately 41 years, and the mean per capita household income was Int$ 896.79 in PPP (Table 1 and Table 2).

A minority of individuals had medical diagnoses of NCDs; however, a statistically significant increase was observed in all chronic diseases analyzed (obesity, T2D, CVD, and HBP) from 2003 to 2015. Regarding health behaviors, few participants adhered to WHO recommendations for leisure-time physical activity, engaged in sedentary behavior, or smoked tobacco products. Trends in health behaviors remained stable throughout the study period (Table 1 and Table 3).

### 3.2. Trends in Prevalence, Duration, and Costs of Health-Related Absenteeism

While only a minority of individuals reported health-related absenteeism, the prevalence, duration, and associated costs increased over the study period (Table 4).

### 3.3. Factors Associated with the Odds and Duration of Health-Related Absenteeism

Considering the associations between health-related absenteeism and cardiometabolic risk factors, positive associations were found for the odds of health-related absenteeism among individuals with a diagnosis of T2D (OR = 1.883, *p* < 0.001), HBP (OR = 1.504, *p* < 0.01), and CVD (OR = 2.744, *p* < 0.001). Similarly, positive associations were observed between diagnosis of these morbidities and the duration of health-related absenteeism: T2D (IRR = 2.290, *p* < 0.05), HBP (IRR = 1.842, *p* < 0.05), and CVD (IRR = 3.128, *p* < 0.05). No statistically significant associations were observed for obesity (Table 5).

Considering lifestyle characteristics, on the one hand, there were no statistically significant associations between the recommended practice of leisure-time physical activity and sedentary behavior about the odds and duration of health-related absenteeism. On the other hand, there was a positive association between smoking tobacco products and the odds of health-related absenteeism (OR = 1.296, *p* < 0.01), and duration of health-related absenteeism (IRR = 2.068, *p* < 0.001) (Table 5).

Regarding the adjustment covariates, factors such as lower income, female sex, older age, private health insurance coverage, and survey years were generally associated with a higher odds or longer duration of health-related absenteeism, whereas having a university degree showed a protective association against longer absences. No statistically significant associations were observed for skin color/ethnicity (Table 5).

### 3.4. Factors Associated with Costs of Health-Related Absenteeism

The analysis of factors associated with the cost of health-related absenteeism showed similar results. A positive association was observed for cardiometabolic risk factors among individuals with a medical diagnosis of T2D (Logit β = 0.328, *p* < 0.05; ME = 55.18, *p* < 0.05), HBP (Logit β = 0.343, *p* < 0.01; ME = 52.13, *p* < 0.05), and CVD (Logit β = 0.854, *p* < 0.01; ME = 62.73, *p* < 0.05), and among individuals with obesity (Logit β = 0.399, *p* < 0.001; ME = 36.45, *p* < 0.05) (Table 6 and Table 7).

Considering lifestyle characteristics, a negative association was observed between health-related absenteeism costs and the recommended level of leisure-time physical activity (GLM β = −0.503, *p* < 0.01; ME = −33.94, *p* < 0.05). On the other hand, a positive association was observed for smoking (GLM β = 0.551, *p* < 0.01; ME = 48.68, *p* < 0.01). No statistically significant associations were observed between sedentary behavior and health-related absenteeism costs (Table 6 and Table 7).

Regarding the adjustment covariates, factors such as higher income, PHI, female sex, older age and being married were associated with higher costs of health-related absenteeism. No statistically significant associations were observed for skin color/ethnicity (Table 6 and Table 7).

## 4. Discussion

The study’s findings indicate that tobacco use and diagnoses of T2D, HBP, and CVD are associated with increased odds, duration, and costs of health-related absenteeism. Obesity was also identified as a cardiometabolic risk factor linked to higher absenteeism costs. Adherence to WHO recommendations for leisure-time physical activity was associated with lower absenteeism costs. Although few studies have examined this association, similar results were reported in two studies from Helsinki, Finland [35,36]. These findings advance understanding of the relationship between recommended leisure-time physical activity and absenteeism costs in a middle-income country context.

Although this study did not find an association between the odds or duration of health-related absenteeism and practicing recommended levels of leisure-time physical activity, previous research in high-income countries such as Belgium [37], Spain [38], Denmark [39,40,41,42], the United Kingdom, France, Finland [12,43,44], and the United States [45] has reported inverse associations. Potential mechanisms underlying these associations may involve improvements in general health indicators through physical activity, as evidenced by both objective [46] and subjective measures [47]. In a study using data from multiple cohorts across European countries, leisure-time physical activity was associated with a greater number of disease-free years, especially for NCDs, among population subgroups with unfavorable socioeconomic backgrounds and pre-existing morbidity risks [46]. In addition, a systematic review and meta-analysis found that leisure-time physical activity is associated with subjective aspects of workers’ general well-being, such as satisfaction with life and positive psychological sensations and effects [47].

Another potential mechanism underlying the association between leisure-time physical activity and absenteeism is the gain in physical and cognitive dexterity resulting from physical activity, leading to improved performance in work activities and physical and mental recovery, especially in response to work-related stress [48,49]. Furthermore, evidence indicates that leisure-time physical activity may help mitigate the negative health effects of sedentary behavior, particularly among white-collar workers, due to the time spent sitting at work [50].

However, differences in population contexts between high-income and low- and middle-income countries are reflected in social determinants of health status and behaviors, particularly in access to health services and lifestyle characteristics [51,52,53]. These environmental factors may explain discrepancies observed in this study compared with previous research in high-income countries on the association between recommended leisure-time physical activity, sedentary behavior, and obesity and the odds and duration of health-related absenteeism.

The determinants of leisure-time physical activity among individuals in the Brazilian population have been analyzed in previous studies, which suggest that socioeconomic characteristics are important predictors of this behavior. Wealthier individuals generally have more opportunities to practice leisure-time physical activity [54]. On the other hand, sedentary behavior during leisure time, especially screen time spent watching television, is concentrated among those with the worst socioeconomic characteristics [55].

Considering the consolidation of the nutritional transition, the current epidemiological stage of obesity in Brazil is characterized by a slightly higher prevalence of the cardiometabolic risk factor among individuals with better socioeconomic indicators, such as household income and education; unlike high-income countries, where obesity is more prevalent among individuals with worse socioeconomic indicators [56]. Thus, the lack of association between the odds and duration of health-related absenteeism, leisure-time physical activity, and obesity, and the simultaneous significant association with the costs of health-related absenteeism, may be explained by associations with socioeconomic characteristics, such as income.

Regarding the adjustment covariates, sociodemographic factors such as income, sex, and age behaved consistently with the existing literature for the Brazilian context. For instance, while higher income and private health insurance were associated with lower odds of absenteeism, they were positively associated with absenteeism costs. This likely reflects higher wages under the Human Capital Approach and greater access to formal medical certification [57,58,59], whereas vulnerable groups or those in informal employment may experience sickness presenteeism due to job insecurity [60,61,62]. Similarly, the higher absenteeism observed among women and older adults aligns with known patterns of healthcare utilization, disease progression, and social determinants such as the dual burden of work and family responsibilities [63,64,65,66,67,68]. Importantly, even after adjusting for these structural determinants and healthcare utilization patterns, lifestyle behaviors and cardiometabolic risk factors remained independent predictors of health-related absenteeism outcomes.

Additional associations identified in this study indicate that cardiometabolic risk factors are significant predictors of the odds, duration, and costs of health-related absenteeism. Similar positive associations between cardiometabolic risk factors, particularly metabolic syndrome and obesity, and the odds and duration of absenteeism have been reported in studies from Japan [69,70] and Portugal [71].

The study identified a significant association between obesity and high blood pressure with absenteeism costs, even after controlling for NCDs such as type 2 diabetes and cardiovascular disease. This finding may indicate that metabolic abnormalities, including high blood pressure or central obesity, affect individuals with a BMI ≤ 30 kg/m^2^. Previous research in Brazil has observed similar risks of metabolic syndrome among individuals with overweight or obesity and those with a healthy weight but presenting metabolic abnormalities [72].

It is worth noting that cardiometabolic risk factors and NCDs are highly sensitive to access and utilization of primary health care and may therefore be preventable through interventions that promote healthier behaviors [3,7]. Public policies aimed at encouraging a more active, less sedentary lifestyle, improving dietary patterns, and minimizing tobacco use may directly support the maintenance of a healthy cardiometabolic profile, thereby helping prevent obesity and associated NCDs [73,74,75].

The use of tobacco is a recognized risk factor for cardiometabolic health [6]. Several studies investigating the association of smoking with health-related work characteristics, like health-related absenteeism, suggest a significant negative impact of smoking on productivity losses due to health reasons [76]. In addition, a previous study conducted in the United States of America’s population estimated a substantial impact of smoking on the costs associated with health-related absenteeism [77]. The results of this study reinforce previous evidence on the subject and suggest the need for sustained strategies to mitigate the negative effects of smoking, particularly regarding potential productivity gains from improved population health.

Furthermore, the substantial increase in absenteeism duration and costs observed in the 2015 survey year may reflect an exogenous epidemiological shock. This period coincided with concurrent outbreaks of Dengue, Zika, and Chikungunya in Brazil [78,79], which cause acute systemic symptoms requiring recovery periods consistent with the observed median absence for that year. However, given the lack of disease-specific absenteeism data, this interpretation remains a plausible contextual hypothesis rather than a direct explanation demonstrated by our study.

A critical consideration when interpreting our findings is the temporal scope of our data, which concludes in 2015. Over the past decade, the global and Brazilian socioeconomic landscapes have undergone profound transformations, most notably precipitated by economic crises and the COVID-19 pandemic. These events triggered a seismic shift in occupational dynamics, particularly the widespread adoption of remote and hybrid work models [80]. In this current post-pandemic context, the traditional operationalization of health-related absenteeism has become increasingly complex [80]. With flexible work arrangements, employees may be more likely to engage in sickness presenteeism [81] rather than formally reporting an absence. Consequently, the threshold for formalizing a sick day may be higher today than it was in 2015, which could alter the frequency and the formal economic valuation of short-term absenteeism [80,81,82].

Furthermore, the COVID-19 pandemic profoundly impacted the very lifestyle behaviors and cardiometabolic risk factors analyzed in this study. Recent evidence suggests a global exacerbation of sedentary behaviors, weight gain, and mental health challenges following the pandemic lockdowns and the transition to screen-heavy remote work [83,84]. Therefore, while the behavioral patterns of taking sick leave may have evolved with hybrid work, the underlying health mechanisms driving productivity losses have likely intensified. In this light, our findings provide a population-level pre-pandemic baseline. Establishing this historical benchmark is essential for future longitudinal studies aiming to quantify how the post-pandemic restructuring of work and the worsening of population cardiometabolic profiles have fundamentally altered the dynamics of health-related absenteeism in middle-income countries.

The present study has certain limitations. First, estimating absenteeism costs using the Human Capital Approach (HCA) based on an individual’s wage fundamentally measures the potential value of lost time, which often overestimates actual productivity losses compared to the Friction Cost Method (FCM) [10,85]. The distinction between these approaches carries profound policy implications; under an FCM framework, actual productivity losses are confined to the friction period, and because the vast majority of health-related absences in our sample are short-term, employers are unlikely to hire temporary replacements. Instead, short-term productivity gaps are typically mitigated by organizational slack: colleagues absorb the workload, or the employee catches up upon returning [10,85].

Consequently, a theoretical application of the FCM would suggest that the actual financial loss to employers for these short absences might be substantially lower, or even negligible, compared to our HCA estimates. Nevertheless, the HCA was retained in this study because it captures the intrinsic value of the workers’ lost health capacity and allows for comparability with the broader international literature. Policymakers should therefore interpret our cost estimates as the upper bound of potential economic value to be recovered through health promotion, rather than strictly as direct financial losses to the labor market.

Second, the potential bias due to self-reported data and the absence of data referring to risky behaviors relevant to health-related absenteeism, like alcohol abuse [86], must be considered a limitation in the findings of the study. Third, the operationalization of the absenteeism variable relies on a broad survey question that includes absences from “school or routine activities” alongside work. While restricting the sample to employed individuals ensures that work is their primary routine activity, this formulation may capture days missed from non-occupational obligations, potentially introducing noise or slightly overestimating the strict number of work days missed.

Another potential limitation of this study is the simultaneous inclusion of lifestyle behaviors and health characteristics in the final multiple regression models. From an inferential perspective, this may result in overadjustment, as health characteristics can act as mediators in the pathway between lifestyle and absenteeism. Consequently, the coefficients for lifestyle factors in our final models represent their direct, mutually adjusted associations rather than their total effects.

However, our supplementary block-wise analysis provides a clear view of these relationships (Tables S1–S3c). Notably, while the effects of lifestyle behaviors are partially attenuated when cardiometabolic conditions are considered, factors such as leisure time physical activity and smoking retain independent associations with absenteeism outcomes. This suggests that their effects may operate through direct pathways beyond just cardiometabolic disease mediation, reinforcing the importance of the fully adjusted analytical approach.

Finally, it is important to note that the cross-sectional design of the ISA-Capital survey impedes causal inference into the associations among lifestyle and cardiometabolic risk factors and the odds, duration, and costs of health-related absenteeism. There is a scarcity of longitudinal surveys with representativeness at the population level encompassing lifestyle, health, demographic and socioeconomic characteristics in the Brazilian population. The results of this study are original contributions with the potential to support the development of future studies with longitudinal design, and to allow evidence-based decision-making in public policies for health promotion and disease prevention, especially in other urban centers in Brazil and other low- and middle-income countries, particularly in Latin America.

## 5. Conclusions

The evidence from this study indicates that modifiable lifestyle factors, cardiometabolic risk factors, and NCDs are significant determinants of the odds, duration, and costs of health-related absenteeism. Smoking tobacco products, high blood pressure, type 2 diabetes, and cardiovascular diseases were associated with higher odds, duration, and costs of absenteeism, while obesity was linked to higher costs. Adherence to recommended leisure-time physical activity was associated with lower absenteeism costs. Promoting active lifestyles, reducing tobacco use, and preventing cardiometabolic risk factors and NCDs may be effective public health strategies to enhance productivity and reduce health-related absenteeism.

## Figures and Tables

**Table 1:** Descriptive statistics of the study variables.

Variables	Categories	Weighted Mean	SE	Min	Max	n
Outcome variables						
Absenteeism	(0 = no/1 = yes)	0.12	0.00	0	1	6504
Duration of absenteeism	(discrete)	1.76	0.20	0	365	6504
Costs of absenteeism	(continuous $PPP)	153.69	24.25	0	61,074.25	6504
Variables of interest						
Obesity	(0 = no/1 = yes)	0.15	0.01	0	1	6504
T2D	(0 = no/1 = yes)	0.06	0.00	0	1	6504
HBP	(0 = no/1 = yes)	0.19	0.01	0	1	6504
CVD	(0 = no/1 = yes)	0.06	0.00	0	1	6504
Recommended leisure PA	(0 = no/1 = yes)	0.23	0.01	0	1	6504
Sedentary behavior	(0 = no/1 = yes)	0.31	0.01	0	1	6504
Smoking	(0 = no/1 = yes)	0.36	0.01	0	1	6504
Control Variables						
Per capita household income	(continuous $PPP)	896.79	41.26	0.91	18,294.86	6504
Low income	(0 = no/1 = yes)	0.31	0.01	0	1	6504
Middle income	(0 = no/1 = yes)	0.33	0.01	0	1	6504
High income	(0 = no/1 = yes)	0.35	0.02	0	1	6504
PHI	(0 = no/1 = yes)	0.31	0.01	0	1	6504
Sex	(0 = male/1 = female)	0.53	0.01	0	1	6504
Age	(discrete)	40.86	0.29	16	95	6504
Skin color/ethnicity	(0 = others/1 = white)	0.60	0.01	0	1	6504
University degree	(0 = others/1 ≥ university)	0.25	0.01	0	1	6504
Marital status	(0 = alone/1 = married)	0.55	0.01	0	1	6504
Survey year	(2003; 2008; 2015)	2009.14	0.14	2003	2015	6504

SE = standard error; Min = minimum; Max = maximum; T2D = type 2 diabetes; HBP = high blood pressure; CVD = cardiovascular diseases; PA = physical activity; PHI = private health insurance; $PPP = purchasing power parity in 2015.

**Table 2:** Socioeconomic and demographic characteristics of the sample by survey year.

Variables	2003	2008	2015	2003–2015	*p*
	(n = 1654)	(n = 2081)	(n = 2769)	(n = 6504)	
Low income	40.74	29.65	27.2	31.98	*
	[36.24, 45.4]	[24.77, 35.03]	[24.24, 30.37]	[29.52, 34.54]	
Middle income	28.28	35.24	35.95	33.47	*
	[24.75, 32.1]	[31.02, 39.69]	[33.22, 38.77]	[31.39, 35.62]	
High income	30.98	35.12	36.85	34.55	
	[25.52, 37.02]	[28.3, 42.6]	[33.1, 40.77]	[31.36, 37.89]	
PHI	33.14	32.63	27.85	31.00	
	[28.99, 37.58]	[27.19, 38.59]	[24.62, 31.33]	[28.43, 33.69]	
Sex	51.91	53.29	52.48	52.58	
	[48.69, 55.12]	[51.04, 55.52]	[50.5, 54.44]	[51.18, 53.98]	
Skin color/ethnicity	67.51	62.23	51.01	59.59	*
	[63.57, 71.22]	[57.18, 67.03]	[47.27, 54.75]	[57.07, 62.07]	
University degree	24.42	23.25	26.69	24.87	
	[20.92, 28.29]	[18.56, 28.71]	[23.31, 30.37]	[22.56, 27.34]	
Marital status	52.42	55.15	57.11	55.08	
	[49.2, 55.61]	[52.11, 58.16]	[54.59, 59.6]	[53.39, 56.77]	
Age categories					
≥16 and <20 years	11.1	7.69	6.76	8.34	*
	[9.89, 12.45]	[6.32, 9.32]	[5.93, 7.71]	[7.66, 9.08]	
≥20 and <40 years	46.95	44.36	43.3	44.72	
	[43.9, 50.02]	[41.26, 47.5]	[40.94, 45.69]	[43.09, 46.36]	
≥40 and <60 years	28.51	33.55	32.94	31.85	*
	[25.71, 31.49]	[31.17, 36.02]	[31, 34.93]	[30.47, 33.26]	
≥60 years	13.44	14.4	17	15.09	*
	[11.8, 15.27]	[12.34, 16.74]	[15.08, 19.11]	[13.96, 16.3]	

Data is presented in weighted percentages of respondents and 95% confidence intervals in brackets. *p*-values were obtained from χ^2^ test to estimate evolution over the period (2003–2015). Statistical significance: * = *p* < 0.05. PHI = private health insurance.

**Table 3:** Lifestyle and health characteristics of the sample by survey year.

Variables	2003	2008	2015	2003–2015	*p*
	(n = 1654)	(n = 2081)	(n = 2769)	(n = 6504)	
Obesity	10.47	13.13	20.33	15.04	*
	[8.291, 13.15]	[11.35, 15.15]	[18.64, 22.14]	[13.91, 16.24]	
T2D	3.98	5.78	7.34	5.83	*
	[3.04, 5.22]	[4.85, 6.87]	[6.37, 8.44]	[5.26, 6.47]	
HBP	14.23	19.83	21.59	18.85	*
	[12.08, 16.7]	[17.95, 21.85]	[19.91, 23.37]	[17.73, 20.03]	
CVD	3.13	4.94	8.64	5.79	*
	[2.15, 4.52]	[3.95, 6.15]	[7.42, 10.03]	[5.12, 6.53]	
Recommended leisure PA	23.74	24.23	22.06	23.28	
	[20.44, 27.38]	[22.06, 26.54]	[19.98, 24.29]	[21.81, 24.81]	
Sedentary behavior	32.03	30.51	30.68	31.02	
	[27.81, 36.55]	[26.55, 34.78]	[28.14, 33.34]	[28.94, 33.17]	
Smoking	37.58	38.07	31.84	35.61	*
	[34.75, 40.49]	[35.09, 41.15]	[29.62, 34.14]	[34.05, 37.19]	

Data is presented in weighted percentages of respondents and 95% confidence intervals in brackets. *p*-values were obtained from χ^2^ test to estimate evolution over the period (2003–2015). Statistical significance: * = *p* < 0.05. T2D = type 2 diabetes; HBP = high blood pressure; CVD = cardiovascular diseases; PA = physical activity; PHI = private health insurance.

**Table 4:** Prevalence of health-related absenteeism, medians of duration and costs of health-related absenteeism, by survey year.

Variables	2003	2008	2015	2003–2015	*p*
	(n = 1654)	(n = 2081)	(n = 2769)	(n = 6504)	
Absenteeism ^a^ (≥1 day of absence)	9.48 [7.89, 11.35]	12.18 [10.61, 13.93]	13.28 [11.83, 14.88]	11.80 [10.89, 12.78]	*
Duration of absenteeism ^b^ (days of absence)	2 [4]	3 [5]	7 [18]	3 [9]	*
Costs of absenteeism ^b^ ($PPP)	13.6 [55.16]	17.43 [186.17]	276.24 [1219.74]	36.92 [216.79]	*

^a^ Data is presented in weighted percentages of respondents and a 95% confidence interval in brackets. *p*-values were obtained from χ^2^ tests to estimate evolution over the period (2003–2015). ^b^ Data is presented in weighted medians and interquartile range in brackets. *p*-values were obtained from a quantile regression model for survey years to estimate evolution in the period (2003–2015). Statistical significance: * = *p* < 0.05. $PPP = purchasing power parity in 2015.

**Table 5:** Factors associated with the odds and duration of health-related absenteeism.

Variables	Categories	Logistic					Negative Binomial				
		OR	SE	Sig.	95%	CI	IRR	SE	Sig.	95%	CI
Variables of interest											
Obesity	1 = yes/0 = no	1.221	0.153		0.953	1.563	1.127	0.310		0.655	1.938
T2D	1 = yes/0 = no	1.883	0.295	***	1.383	2.563	2.290	0.871	*	1.082	4.843
HBP	1 = yes/0 = no	1.504	0.223	**	1.123	2.014	1.842	0.533	*	1.042	3.254
CVD	1 = yes/0 = no	2.744	0.709	***	1.648	4.566	3.128	1.379	*	1.312	7.451
Recommended leisure PA	1 = yes/0 = no	0.946	0.109		0.753	1.186	0.802	0.161		0.539	1.191
Sedentary behavior	1 = yes/0 = no	1.083	0.122		0.867	1.353	0.946	0.188		0.639	1.398
Smoking	1 = yes/0 = no	1.296	0.115	**	1.088	1.543	2.068	0.426	***	1.379	3.102
Control variables											
Income	Ref. = lower income										
Middle income	1 = yes/0 = no	0.657	0.068	***	0.535	0.804	0.495	0.121	**	0.306	0.799
High income	1 = yes/0 = no	0.737	0.091	*	0.577	0.940	0.529	0.146	*	0.306	0.912
PHI	1 = yes/0 = no	1.283	0.126	*	1.058	1.555	1.732	0.417	*	1.078	2.780
Sex	1 = female/0 = male	1.442	0.127	***	1.211	1.715	1.416	0.239	*	1.015	1.974
Age	Years	0.999	0.003		0.993	1.003	1.012	0.005	*	1.002	1.022
Skin color	1 = white/0 = others	0.993	0.101		0.813	1.211	1.082	0.213		0.733	1.594
University degree	1 ≥ university/0 = others	0.832	0.126		0.617	1.121	0.588	0.142	*	0.365	0.944
Marital status	1 = married/0 = alone	0.861	0.082		0.714	1.037	0.715	0.158		0.461	1.105
Year of survey	Ref. = 2003										
2008	1 = 2008/0 = no	1.319	0.165	*	1.030	1.689	1.295	0.269		0.859	1.950
2015	1 = 2015/0 = no	1.476	0.180	**	1.160	1.878	7.214	1.766	***	4.455	11.684
Constant		0.092	0.015	***	0.066	0.126	0.310	0.098	***	0.166	0.578
n		6504					6504				

OR = odds ratio; IRR = incidence rate ratio; SE = robust standard error. T2D = type 2 diabetes; HBP = high blood pressure; CVD = cardiovascular diseases; PA = physical activity; PHI = private health insurance. Sig. = *** *p* < 0.001; ** *p* < 0.01; * *p* < 0.05. Estimates were calculated considering the complex survey design.

**Table 6:** Factors associated with health-related absenteeism costs.

Variables	Categories	Logit					GLM				
		β	SE	Sig.	95%	CI	β	SE	Sig.	95%	CI
Variables of interest											
Obesity	1 = yes/0 = no	0.399	0.110	***	0.183	0.615	0.131	0.229		−0.321	0.582
T2D	1 = yes/0 = no	0.328	0.133	*	0.065	0.590	0.418	0.311		−0.195	1.030
HBP	1 = yes/0 = no	0.343	0.121	**	0.104	0.580	0.369	0.291		−0.204	0.942
CVD	1 = yes/0 = no	0.854	0.253	**	0.356	1.351	0.092	0.316		−0.530	0.714
Recommended leisure PA	1 = yes/0 = no	0.107	0.092		−0.073	0.287	−0.503	0.191	**	−0.879	−0.126
Sedentary behavior	1 = yes/0 = no	0.142	0.091		−0.037	0.320	0.169	0.155		−0.136	0.474
Smoking	1 = yes/0 = no	0.061	0.072		−0.080	0.203	0.551	0.184	**	0.188	0.912
Control variables											
Income	Ref. = lower income										
Middle income	1 = yes/0 = no	−0.496	0.098	***	−0.688	−0.302	0.487	0.200	*	0.092	0.880
High income	1 = yes/0 = no	−0.512	0.111	***	−0.730	−0.293	1.903	0.248	***	1.413	2.392
PHI	1 = yes/0 = no	0.166	0.097		−0.025	0.357	0.439	0.186	*	0.072	0.806
Sex	1 = female/0 = male	0.199	0.072	**	0.056	0.341	0.069	0.161		−0.248	0.386
Age	Years	0.014	0.002	***	0.009	0.018	0.007	0.005		−0.003	0.016
Skin color	1 = white/0 = others	0.030	0.085		−0.137	0.197	0.263	0.183		−0.097	0.624
University degree	1 ≥ university/0 = others	−0.050	0.130		−0.306	0.207	−0.368	0.243		−0.846	0.110
Marital status	1 = married/0 = alone	−0.400	0.080		−0.556	−0.242	0.412	0.170	*	0.078	0.745
Year of survey	Ref. = 2003										
2008	1 = 2008/0 = no	−0.001	0.105		−0.210	0.207	0.384	0.206		−0.021	0.791
2015	1 = 2015/0 = no	−1.390	0.144	***	−1.238	−0.794	2.633	0.221	***	2.196	3.069
Constant		−1.390	0.145	***	−1.675	−1.105	3.111	0.282	***	2.556	3.665
n		6504					6504				

GLM = generalized linear model; β = coefficient; SE = robust standard error; T2D = type 2 diabetes; HBP = high blood pressure; CVD = cardiovascular diseases; PA = physical activity; PHI = private health insurance. Sig. = *** *p* < 0.001; ** *p* < 0.01; * *p* < 0.05. Estimates were calculated considering the complex survey design.

**Table 7:** Average marginal effects from the two-part regression model of factors associated with health-related absenteeism costs.

Variables	Categories	ME				
		dy/dx	SE	Sig.	95%	CI
Variables of interest						
Obesity	1 = yes/0 = no	36.45	18.32	*	0.37	72.51
T2D	1 = yes/0 = no	55.18	27.11	*	1.78	108.56
HBP	1 = yes/0 = no	52.13	26.20	*	0.55	103.71
CVD	1 = yes/0 = no	62.73	26.83	*	9.90	115.55
Recommended leisure PA	1 = yes/0 = no	−33.94	16.43	*	−66.29	−1.58
Sedentary behavior	1 = yes/0 = no	22.89	13.36		−3.41	49.19
Smoking	1 = yes/0 = no	48.68	16.82	**	15.57	81.79
Control variables						
Income	Ref. = lower income					
Middle income	1 = yes/0 = no	4.89	9.75		−14.31	24.09
High income	1 = yes/0 = no	162.50	41.58	***	80.63	244.37
PHI	1 = yes/0 = no	46.41	15.73	**	15.43	77.39
Sex	1 = female/0 = male	18.49	14.06		−9.18	46.16
Age	Years	1.45	0.43	**	0.60	2.29
Skin color	1 = white/0 = others	23.32	16.14		−8.45	55.08
University degree	1 ≥ university/0 = others	−33.06	21.01		−74.42	8.30
Marital status	1 = married/0 = alone	7.59	15.14		−22.22	37.40
Year of survey	Ref. = 2003					
2008	1 = 2008/0 = no	16.70	9.10		−1.21	34.63
2015	1 = 2015/0 = no	182.03	41.08	***	101.13	262.92
n		6504				

ME = marginal effects; dy/dx = marginal effects; SE = robust standard error; T2D = type 2 diabetes; HBP = high blood pressure; CVD = cardiovascular diseases; PA = physical activity; PHI = private health insurance. Sig. = *** *p* < 0.001; ** *p* < 0.01; * *p* < 0.05. Estimates were calculated considering complex survey design.

## Data Availability

The data presented in this study are available upon request from the corresponding author due to privacy and institutional restrictions.

## Supplementary Materials

The following supporting information can be downloaded at https://www.mdpi.com/article/10.3390/healthcare14101260/s1, Table S1. Block wise logistic regression model for factors associated with the likelihood of health-related absenteeism. Table S2. Block wise negative binomial regression model for factors associated with the duration of health-related absenteeism. Table S3. (a). Logit regression from block wise two-part model of factors associated with health-related absenteeism costs. (b). Generalized linear model from block wise two-part model of factors associated with health-related absenteeism costs. (c). Average Marginal effects from block wise two-part model of factors associated with health-related absenteeism costs. Table S4. Variance inflation factors of the covariates of the study. Table S5. Spearman rank correlation matrix of the covariates used in the statistical models of the study.

## Abbreviations

The following abbreviations are used in this manuscript:

NCDs	Non-communicable disease
ISA-Capital	São Paulo Health Survey
GLM	Generalized linear model
ME	Average marginal effects
HBP	High blood pressure
T2D	Type 2 diabetes
CVD	Cardiovascular disease
IPCA	Broad consumer price index
IBGE	Brazilian institute of geography and statistics
PPP	Purchase power parity
WHO	World Health Organization
BMI	Body mass index
OR	Odds ratio
IRR	Incidence rate ratio
SE	Robust standard error
PA	Physical activity
PHI	Private health insurance
VIF	Variance inflation factor
HCA	Human Capital Approach
FCM	Friction Cost Method
