# Supplementary material for: Factors Associated with the Odds, Duration, and Costs of Health-Related Absenteeism: A Population-Based Study in São Paulo City, Brazil
Source: Healthcare (Basel). 2026 May 7;14(10):1260. doi: 10.3390/healthcare14101260 (PMC13206122; doi:10.3390/healthcare14101260)
Supplement: Supplementary file 1 [file healthcare-14-01260-s001.zip › healthcare-4170582-supplementary.pdf]

Table S1. Block wise logistic regression model for factors associated with the likelihood of health-related absenteeism.

Variables	Categories	Block 1 (socioeconomic and demographic controls)					Block 2 (Block 1 + lifestyle characteristics)					Block 3 (block 2 + health characteristics)				
		**OR**	**SE**	**P-value**	**95%**	**CI**	**OR**	**SE**	**P-value**	**95%**	**CI**	**OR**	**SE**	**P-value**	**95%**	**CI**
Income	Ref. = lower income															
Middle income	1=yes/0=no	0.652	0.067	0.000	0.533	0.797	0.654	0.067	0.000	0.535	0.801	0.657	0.068	0.000	0.536	0.805
High income	1=yes/0=no	0.724	0.090	0.010	0.567	0.924	0.725	0.090	0.010	0.568	0.926	0.737	0.091	0.014	0.577	0.940
PHI	1=yes/0=no	1.304	0.128	0.007	1.075	1.581	1.321	0.129	0.005	1.090	1.600	1.283	0.126	0.011	1.058	1.556
Sex	1=female/0=male	1.405	0.125	0.000	1.179	1.673	1.455	0.127	0.000	1.225	1.728	1.442	0.127	0.000	1.212	1.715
Age	Years	1.006	0.002	0.014	1.001	1.010	1.005	0.002	0.043	1.000	1.009	0.999	0.003	0.596	0.993	1.004
Skin color	1=white/0=others	0.996	0.102	0.966	0.815	1.217	0.993	0.102	0.943	0.811	1.214	0.993	0.101	0.943	0.813	1.212
High education	1>=university/0=others	0.798	0.122	0.142	0.591	1.079	0.799	0.121	0.139	0.593	1.076	0.832	0.126	0.226	0.617	1.122
Marital status	1=married/0=alone	0.868	0.080	0.126	0.724	1.041	0.855	0.081	0.100	0.710	1.031	0.861	0.082	0.116	0.714	1.038
Year	Ref. = 2003															
2008	1=2008/0=no	1.365	0.172	0.014	1.065	1.749	1.366	0.171	0.013	1.068	1.747	1.319	0.166	0.028	1.030	1.689
2015	1=2015/0=no	1.557	0.190	0.000	1.224	1.980	1.585	0.193	0.000	1.246	2.015	1.476	0.181	0.002	1.160	1.878
Constant		0.089	0.013	0.000	0.067	0.118										
Recommended leisure PA	1=yes/0=no						0.952	0.109	0.669	0.760	1.193	0.946	0.109	0.630	0.754	1.187
Sedentary habits	1=yes/0=no						1.091	0.124	0.444	0.872	1.364	1.083	0.122	0.478	0.867	1.353
Smoking	1=yes/0=no						1.302	0.116	0.003	1.092	1.552	1.296	0.115	0.004	1.088	1.543
Constant							0.081	0.013	0.000	0.059	0.110					
Obesity	1=yes/0=no											1.221	0.153	0.114	0.953	1.563
T2D	1=yes/0=no											1.883	0.295	0.000	1.383	2.563
HBP	1=yes/0=no											1.504	0.223	0.006	1.124	2.014
CVD	1=yes/0=no											2.744	0.710	0.000	1.648	4.567
Constant												0.092	0.015	0.000	0.067	0.127
N				6504					6504					6504		

OR = Odds Ratio; SE = robust standard error; T2D=type 2 diabetes mellitus; HBP = high blood pressure; CVD = cardiovascular diseases; PA = physical activity; PHI = private health insurance; N = sample size.

Table S2. Block wise negative binomial regression model for factors associated with the duration of health-related absenteeism.

Variables	Categories	Block 1 (socioeconomic and demographic controls)					Block 2 (Block 1 + lifestyle characteristics)					Block 3 (block 2 + health characteristics)				
		IRR	SE	P-value	95%	CI	IRR	SE	P-value	95%	CI	IRR	SE	P-value	95%	CI
Income	Ref. = lower income															
Middle income	1=yes/0=no	0.462	0.123	0.004	0.274	0.779	0.418	0.106	0.001	0.254	0.688	0.495	0.121	0.004	0.306	0.799
High income	1=yes/0=no	0.461	0.139	0.011	0.255	0.833	0.431	0.122	0.003	0.247	0.752	0.529	0.146	0.022	0.306	0.912
PHI	1=yes/0=no	1.708	0.461	0.048	1.004	2.905	1.787	0.415	0.013	1.131	2.823	1.732	0.417	0.023	1.079	2.781
Sex	1=female/0=male	1.113	0.205	0.561	0.775	1.598	1.363	0.241	0.080	0.963	1.930	1.416	0.239	0.040	1.015	1.974
Age	Years	1.019	0.006	0.001	1.008	1.030	1.019	0.005	0.000	1.009	1.029	1.012	0.005	0.018	1.002	1.022
Skin color	1=white/0=others	1.105	0.227	0.628	0.738	1.655	1.059	0.207	0.769	0.721	1.555	1.082	0.213	0.691	0.734	1.595
High education	1>=university/0=others	0.633	0.169	0.088	0.374	1.071	0.597	0.136	0.025	0.381	0.936	0.588	0.142	0.028	0.366	0.945
Marital status	1=married/0=alone	0.752	0.169	0.206	0.482	1.171	0.659	0.151	0.069	0.420	1.033	0.715	0.158	0.131	0.462	1.106
Year	Ref. = 2003															
2008	1=2008/0=no	1.155	0.304	0.586	0.687	1.940	1.406	0.320	0.135	0.899	2.200	1.295	0.269	0.215	0.860	1.951
2015	1=2015/0=no	5.840	1.502	0.000	3.519	9.691	7.103	1.694	0.000	4.442	11.359	7.215	1.767	0.000	4.455	11.684
Constant		0.487	0.202	0.084	0.215	1.101										
Recommended leisure PA	1=yes/0=no						0.738	0.150	0.135	0.495	1.100	0.802	0.161	0.273	0.539	1.191
Sedentary habits	1=yes/0=no						0.930	0.188	0.721	0.626	1.384	0.946	0.188	0.780	0.640	1.399
Smoking	1=yes/0=no						2.022	0.418	0.001	1.346	3.036	2.068	0.426	0.000	1.379	3.102
Constant							0.348	0.131	0.005	0.166	0.730					
Obesity	1=yes/0=no											1.127	0.310	0.665	0.655	1.938
T2D	1=yes/0=no											2.290	0.871	0.030	1.082	4.844
HBP	1=yes/0=no											1.842	0.533	0.036	1.043	3.255
CVD	1=yes/0=no											3.128	1.379	0.010	1.313	7.451
Constant												0.310	0.098	0.000	0.166	0.579
N				6504					6504					6504		

IRR = Incidence Rate Ratio; SE = robust standard error; T2D=type 2 diabetes mellitus; HBP = high blood pressure; CVD = cardiovascular diseases; PA = physical activity; PHI = private health insurance; N = sample size.

Table S3a. Logit regression from block wise two-part model of factors associated with health-related absenteeism costs.

Variables	Categories	Logit, Block 1 (socioeconomic and demographic controls)					Logit, Block 2 (Block 1 + lifestyle characteristics)					Logit, Block 3 (block 2 + health characteristics)				
		**b**	**SE**	**P-value**	**95%**	**CI**	**b**	**SE**	**P-value**	**95%**	**CI**	**b**	**SE**	**P-value**	**95%**	**CI**
Income	Ref. = lower income															
Middle income	1=yes/0=no	-0.487	0.098	0.000	-0.680	-0.295	-0.492	0.098	0.000	-0.685	-0.300	-0.496	0.098	0.000	-0.689	-0.303
High income	1=yes/0=no	-0.505	0.110	0.000	-0.722	-0.288	-0.520	0.111	0.000	-0.738	-0.302	-0.512	0.111	0.000	-0.731	-0.294
PHI	1=yes/0=no	0.192	0.097	0.049	0.001	0.383	0.189	0.097	0.053	-0.002	0.381	0.166	0.097	0.089	-0.025	0.357
Sex	1=female/0=male	0.185	0.073	0.011	0.042	0.327	0.208	0.072	0.004	0.067	0.349	0.199	0.072	0.006	0.057	0.341
Age	Years	0.018	0.002	0.000	0.015	0.022	0.019	0.002	0.000	0.015	0.022	0.014	0.002	0.000	0.010	0.019
Skin color	1=white/0=others	0.030	0.085	0.723	-0.137	0.198	0.026	0.085	0.764	-0.142	0.193	0.030	0.085	0.725	-0.138	0.198
High education	1>=university/0=others	-0.054	0.134	0.684	-0.317	0.209	-0.078	0.131	0.550	-0.337	0.180	-0.050	0.130	0.704	-0.306	0.207
Marital status	1=married/0=alone	-0.397	0.078	0.000	-0.550	-0.244	-0.389	0.079	0.000	-0.545	-0.232	-0.400	0.080	0.000	-0.557	-0.243
Year	Ref. = 2003															
2008	1=2008/0=no	0.029	0.104	0.782	-0.176	0.234	0.029	0.105	0.780	-0.177	0.236	-0.001	0.106	0.989	-0.210	0.207
2015	1=2015/0=no	-0.941	0.111	0.000	-1.159	-0.723	-0.934	0.111	0.000	-1.152	-0.716	-1.016	0.113	0.000	-1.238	-0.794
Constant		-1.373	0.135	0.000	-1.638	-1.108										
Recommended leisure PA	1=yes/0=no						0.107	0.093	0.253	-0.076	0.289	0.107	0.092	0.245	-0.074	0.287
Sedentary habits	1=yes/0=no						0.155	0.091	0.090	-0.025	0.335	0.142	0.091	0.120	-0.037	0.321
Smoking	1=yes/0=no						0.063	0.073	0.391	-0.081	0.206	0.061	0.072	0.395	-0.081	0.204
Constant							-1.476	0.140	0.000	-1.751	-1.201					
Obesity	1=yes/0=no											0.399	0.110	0.000	0.183	0.615
T2D	1=yes/0=no											0.328	0.133	0.014	0.066	0.591
HBP	1=yes/0=no											0.343	0.121	0.005	0.104	0.581
CVD	1=yes/0=no											0.854	0.253	0.001	0.356	1.352
Constant												-1.390	0.145	0.000	-1.675	-1.106
N				6504					6504					6504		

b = coefficient; SE = robust standard error; T2D=type 2 diabetes mellitus; HBP = high blood pressure; CVD = cardiovascular diseases; PA = physical activity; PHI = private health insurance; N = sample size.

Table S3b. Generalized linear model from block wise two-part model of factors associated with health-related absenteeism costs.

Variables	Categories	GLM, Block 1 (socioeconomic and demographic controls)					GLM, Block 2 (Block 1 + lifestyle characteristics)					GLM, Block 3 (block 2 + health characteristics)				
		b	SE	P-value	95%	CI	b	SE	P-value	95%	CI	b	SE	P-value	95%	CI
Income	Ref. = lower income															
Middle income	1=yes/0=no	0.484	0.222	0.030	0.046	0.922	0.459	0.206	0.027	0.054	0.863	0.487	0.200	0.016	0.093	0.881
High income	1=yes/0=no	1.827	0.285	0.000	1.267	2.388	1.856	0.256	0.000	1.351	2.360	1.903	0.248	0.000	1.414	2.392
PHI	1=yes/0=no	0.376	0.209	0.073	-0.036	0.787	0.463	0.188	0.015	0.092	0.833	0.439	0.186	0.019	0.073	0.806
Sex	1=female/0=male	0.042	0.173	0.808	-0.298	0.382	0.052	0.166	0.756	-0.275	0.378	0.069	0.161	0.669	-0.249	0.387
Age	Years	0.012	0.005	0.022	0.002	0.023	0.011	0.005	0.036	0.001	0.021	0.007	0.005	0.181	-0.003	0.016
Skin color	1=white/0=others	0.277	0.196	0.159	-0.109	0.664	0.232	0.182	0.204	-0.127	0.590	0.263	0.183	0.152	-0.098	0.624
							-					-				
High education	1>=university/0=others	-0.304	0.259	0.242	-0.813	0.206	0.393	0.247	0.113	-0.879	0.094	0.368	0.243	0.132	-0.846	0.111
Marital status	1=married/0=alone	0.407	0.203	0.046	0.007	0.806	0.356	0.178	0.046	0.006	0.707	0.412	0.170	0.016	0.078	0.746
Year	Ref. = 2003															
2008	1=2008/0=no	0.278	0.244	0.257	-0.203	0.759	0.384	0.222	0.084	-0.052	0.821	0.385	0.206	0.063	-0.022	0.791
2015	1=2015/0=no	2.518	0.253	0.000	2.019	3.016	2.613	0.228	0.000	2.165	3.062	2.633	0.222	0.000	2.197	3.070
Constant		3.283	0.342	0.000	2.610	3.956										
							-					-				
Recommended leisure PA	1=yes/0=no						0.545	0.192	0.005	-0.922	-0.167	0.503	0.191	0.009	-0.880	-0.126
Sedentary habits	1=yes/0=no						0.188	0.158	0.233	-0.122	0.499	0.169	0.155	0.277	-0.137	0.475
Smoking	1=yes/0=no						0.545	0.184	0.003	0.184	0.907	0.551	0.184	0.003	0.188	0.913
Constant							3.139	0.335	0.000	2.480	3.798					
Obesity	1=yes/0=no											0.131	0.229	0.570	-0.321	0.582
T2D	1=yes/0=no											0.418	0.311	0.181	-0.195	1.031
HBP	1=yes/0=no											0.369	0.291	0.206	-0.204	0.943
CVD	1=yes/0=no											0.092	0.316	0.771	-0.531	0.715
Constant												3.111	0.282	0.000	2.556	3.666
N				6504					6504					6504		

GLM = generalized linear model; b = coefficient; SE = robust standard error; T2D=type 2 diabetes mellitus; HBP = high blood pressure; CVD = cardiovascular diseases; PA = physical activity; PHI = private health insurance; N = sample size.

Table S3c. Average Marginal effects from block wise two-part model of factors associated with health-related absenteeism costs.

Variables	Categories	ME, Block 1 (socioeconomic and demographic controls)					ME, Block 2 (Block 1 + lifestyle characteristics)					ME, Block 3 (block 2 + health characteristics)				
		**dy/dx**	**SE**	**P-value**	**95%**	**CI**	**dy/dx**	**SE**	**P-value**	**95%**	**CI**	**dy/dx**	**SE**	**P-value**	**95%**	**CI**
Income	Ref. = lower income															
Middle income	1=yes/0=no	5.71	12.17	0.640	-18.26	29.67	3.83	10.49	0.715	-16.82	24.48	4.89	9.75	0.616	-14.31	24.09
High income	1=yes/0=no	165.19	47.30	0.001	72.05	258.33	159.27	42.04	0.000	76.50	242.04	162.50	41.58	0.000	80.63	244.37
PHI	1=yes/0=no	46.68	19.00	0.015	9.27	84.09	50.91	16.39	0.002	18.65	83.17	46.41	15.73	0.003	15.43	77.39
Sex	1=female/0=male	16.67	16.33	0.308	-15.48	48.81	18.02	14.66	0.220	-10.85	46.89	18.49	14.06	0.189	-9.18	46.16
Age	Years	2.39	0.50	0.000	1.40	3.38	2.11	0.46	0.000	1.20	3.02	1.45	0.43	0.001	0.61	2.30
Skin color	1=white/0=others	26.60	19.28	0.169	-11.36	64.55	20.91	16.32	0.201	-11.23	53.05	23.32	16.14	0.150	-8.45	55.09
High education	1>=university/0=others	-30.63	24.20	0.207	-78.28	17.01	-37.76	21.94	0.086	-80.96	5.44	-33.06	21.01	0.117	-74.42	8.30
Marital status	1=married/0=alone	8.02	19.15	0.676	-29.68	45.72	3.94	15.93	0.805	-27.42	35.29	7.59	15.14	0.617	-22.22	37.40
Year	Ref. = 2003															
2008	1=2008/0=no	14.23	11.32	0.210	-8.06	36.52	17.85	9.90	0.072	-1.64	37.35	16.71	9.10	0.068	-1.22	34.64
2015	1=2015/0=no	195.17	44.18	0.000	108.18	282.15	192.49	42.33	0.000	109.16	275.83	182.03	41.09	0.000	101.14	262.93
Recommended leisure PA	1=yes/0=no						-38.19	16.83	0.024	-71.33	-5.06	-33.94	16.43	0.040	-66.29	-1.58
Sedentary habits	1=yes/0=no						25.89	13.94	0.064	-1.56	53.34	22.89	13.36	0.088	-3.42	49.20
Smoking	1=yes/0=no						49.39	16.88	0.004	16.16	82.62	48.68	16.82	0.004	15.57	81.80
Obesity	1=yes/0=no											36.45	18.32	0.048	0.37	72.52
T2D	1=yes/0=no											55.18	27.11	0.043	1.79	108.56
HBP	1=yes/0=no											52.13	26.20	0.048	0.56	103.71
CVD	1=yes/0=no											62.73	26.83	0.020	9.90	115.55
N				6504					6504					6504		

ME = marginal effects; dy/dx = marginal effect; SE = robust standard error; T2D=type 2 diabetes mellitus; HBP = high blood pressure; CVD = cardiovascular diseases; PA = physical activity; PHI = private health insurance; N = sample size.

Table S4. Variance inflation factors of the covariates of the study.

Variables	VIF	1/VIF
Obesity	1.09	0.915258
T2D	1.13	0.881813
HBP	1.22	0.821722
CVD	1.03	0.975416
Leisure PA	1.04	0.959355
Sedentary habits	1.05	0.954152
Smoking	1.07	0.937136
Income (ref. = low)		
Middle income	1.43	0.697645
High income	1.88	0.532508
PHI	1.23	0.815031
Sex	1.06	0.944106
Age	1.41	0.708171
Skin color	1.12	0.893135
High education	1.35	0.739011
Marital status	1.08	0.923751
Survey year (ref. = 2003)		
2008	1.46	0.686532
2015	1.53	0.651615
Mean VIF	1.25	

VIF = variance inflation factor; T2D = type 2 diabetes; HBP = high blood pressure; CVD = cardiovascular disease; PA = physical activity; PHI = private health insurance.

Table S5. Spearman rank correlation matrix of the covariates used in the statistical models of the study.

	Obesity	T2D	HBP	CVD	Leisure PA	Sedentary behavior	Smoking	Income	PHI	Sex	Age	Skin color	High education	Marital status	Survey year
Obesity	1.000														
T2D	0.119	1.000													
HBP	-0.180	-0.133	1.000												
CVD	-0.069	-0.051	-0.065	1.000											
Leisure PA	-0.045	-0.024	-0.012	0.008	1.000										
Sedentary behavior	0.028	0.009	-0.020	0.007	-0.021	1.000									
Smoking	0.020	0.043	0.055	0.010	-0.041	-0.008	1.000								
Income	0.044	0.012	0.004	0.004	0.071	0.081	-0.019	1.000							
PHI	0.031	0.035	0.015	0.031	0.048	0.069	-0.053	0.330	1.000						
Sex	0.055	0.036	0.044	-0.026	-0.115	-0.029	-0.168	-0.035	0.017	1.000					
Age	0.129	0.288	0.324	0.053	-0.089	-0.057	0.166	0.141	0.088	0.055	1.000				
Skin color	0.015	0.033	0.005	0.019	0.020	0.040	-0.003	0.183	0.187	0.042	0.141	1.000			
High education	-0.002	-0.057	-0.087	-0.008	0.106	0.113	-0.044	0.368	0.229	-0.007	-0.088	0.141	1.000		
Marital status	0.080	0.024	0.069	0.011	-0.044	-0.097	0.151	0.046	0.062	-0.127	0.225	0.018	-0.027	1.000	
Survey year	0.100	0.027	-0.015	0.033	-0.025	-0.018	-0.042	0.125	-0.053	0.052	-0.018	-0.134	0.074	0.045	1.000

T2D = type 2 diabetes; HBP = high blood pressure; CVD = cardiovascular disease; PA = physical activity; PHI = private health insurance.
